# Prevalence of pathogenic *Klebsiella pneumoniae* based on PCR capsular typing harbouring carbapenemases encoding genes in Uganda tertiary hospitals

**DOI:** 10.1186/s13756-021-00923-w

**Published:** 2021-03-18

**Authors:** Kenneth Ssekatawa, Denis K. Byarugaba, Jesca L. Nakavuma, Charles D. Kato, Francis Ejobi, Robert Tweyongyere, Wampande M. Eddie

**Affiliations:** 1 College of Veterinary Medicine, Animal Resources and Biosecurity, Makerere University, P. O. Box 7062, Kampala, Uganda; 2 Department of Biochemistry, Faculty of Biomedical Sciences, Kampala International University-Western Campus, P. O. Box 71, Bushenyi, Uganda; 3 Africa Center Excellence in Materials Product Development and Nanotechnology (MAPRONANO ACE), College of Engineering Design Art and Technology, Makerere University, P. O. Box 7062, Kampala, Uganda

**Keywords:** Carbapenem resistance, *Klebsiella pneumoniae* PCR capsular typing, Virulent factors

## Abstract

**Background:**

*Klebsiella pneumoniae* is an opportunistic pathogen that has been implicated as one of commonest cause of hospital and community acquired infections. The *K. pneumoniae* infections have considerably contributed to morbidity and mortality in patients with protracted ailments. The capacity of *K. pneumoniae* to cause diseases depends on the presence of an array virulence factors. Coexistence and expression of virulence factors and genetic determinants of antibiotic resistance complicates treatment outcomes. Thus, emergence of pathogenic MDR *K. pneumoniae* poses a great threat to the healthcare system. However, the carriage of antibiotic resistance among pathogenic *K. pneumoniae* is yet to be investigated in Uganda. We sought to investigate the carbapenem resistance profiles and pathogenic potential based on capsular serotypes of *K. pneumoniae* clinical isolates.

**Methods:**

This was a cross sectional study involving use of archived *Klebsiella pneumoniae* isolates collected between January and December, 2019 at four tertiary hospitals in Uganda. All isolates were subject to antimicrobial susceptibility assays to determine phenotypic antibiotic resistance, pentaplex PCR to detect carbapenemases encoding genes and heptaplex PCR to identify capsular serotypes K1, K2, K3, K5, K20, K54 and K57.

**Results:**

The study found an overall phenotypic carbapenem resistance of 23.3% (53/227) and significantly higher genotypic resistance prevalence of 43.1% (98/227). Over all, the most prevalent gene was *bla*_OXA-48-like_ (36.4%), followed by *bla*_IMP-type_ (19.4%), *bla*_VIM-type_ (17.1%), *bla*_KPC-type_ (14.0%) and *bla*_NDM-type_ (13.2%). *bla*_VIM-type_ and *bla*_OXA-48-like_ conferred phenotypic resistance in all isolates and 38.3% of isolates that harbored them respectively. Capsular multiplex PCR revealed that 46.7% (106/227) isolates were pathogenic and the predominantly prevalent pathotype was K5 (18.5%) followed by K20 (15.1%), K3 (7.1%), K2 (3.1%) and K1 (2.2%). Of the 106 capsular serotypes, 37 expressed phenotypic resistance; thus, 37 of the 53 carbapenem resistant *K. pneumoniae* were pathogenic.

**Conclusion:**

The high prevalence of virulent and antibiotic resistant *K. pneumoniae* among clinical isolates obtained from the four tertiary hospital as revealed by this study pose a great threat to healthcare. Our findings underline the epidemiological and public health risks and implications of this pathogen.

## Background

The growing prevalence of antibiotic-resistant clinical bacterial isolates is one of the main burdens to the healthcare systems worldwide [1, 2]. Knowledge of antibiotic resistance genetic determinants is critical in thwarting the emergence and spread of multidrug-resistant (MDR) bacteria. Of great concern, is the spread of MDR strains of pathogenic *Klebsiella pneumoniae*, the Gram-negative bacteria that cause healthcare associated infections (HAI), community acquired infections, urinary tract infections (UTI) and wound infections. *K*. *pneumoniae* can harbor and express beta lactamases, most importantly carbapenemases capable of hydrolyzing newer carbapenem drugs used in the treatment of MDR bacterial infections [3–5].

An array of virulent factors responsible for pathogenesis such as endotoxins, capsules, iron-scavenging systems, siderophores and adhesions can be expressed by *K. pneumoniae*. A capsule is a vital virulence factor, because it confers two pathogenic mechanisms; shielding the invading bacteria from phagocytosis, and neutralizing the host immune response [6]. *Klebsiella* capsular serotyping (K typing) differentiates *K. pneumoniae* into approximately 77 K types [7]. Several capsular (K) types, predominantly K1, K2, K54, K57, K20, and K5, are frequently linked to community-acquired invasive septicemia, pyogenic liver abscess syndrome and pneumonia [8, 9]. K3 is the usual cause of rhinoscleroma [10]. Pathogen survival requires the acquisition of drug resistance and virulent factors [11] and the acquired traits have been postulated to play an important part in the pathogenesis of *K. pneumoniae* infections [12].

Molecular capsular typing is presently the main technique employed in characterization of *K. pneumoniae* isolates and demonstrates excellent reproducibility in distinguishing clinical isolates [13]. Multiplex PCRs for detecting of the capsule repeat unit polymerase Wzy genes can be utilized for capsule typing of *K. pneumoniae* [14, 15]. Therefore, virulence factors encoding genes can be used to characterize the different pathotypes of *K. pneumonia.* In spite of this, the carbapenem resistance profiles of pathogenic *K. pneumoniae* in Uganda are yet to be documented. Thus, to obtain insights into this, we characterized the carbapenem genetic resistance determinants among the pathogenic *Klebsiella pneumoniae* clinical isolates collected from four main referral hospitals in Uganda.

## Materials and methods

### Bacterial strains

The study used 227 out of 284 archived MDR *Klebsiella pneumoniae* isolated between January and December, 2019 from clinical specimens in the Microbiology Laboratories of Mulago National Referral Hospital (MNRH) located in the central region 03381°N, 32.5761°E, Mbale Regional Referral Hospital (MRRH) in the Eastern region 1.0766°N, 34.1768°E, Mbarara Regional Referral Hospital (MBRRH),Western region 0.6171° S, 30.6577° E and Kampala International University Teaching Hospital (KIU-TH), Western region, 0.5468° S, 30.1387° E, Table 1.The isolates were initially assayed and confirmed to be resistant to several antibiotics.

**Table 1:** Genes and their primers sequences for molecular characterization of K. pneumoniae

Target genes	Primer sequence	Ampilicon size (Bp)	References
khe	F: TGATTGCATTCGCCACTGG R: GGTCAACCCAACGATCCTG	428	Neuberger et al. [53]
WzyK1	F: GGTGCTCTTTACATCATTGC R: GCAATGGCCATTTGCGTTAG	1283	Turton et al. [54]
WzyK2	F: GACCCGATATTCATACTTGACAGAG R: CCTGAAGTAAAATCGTAAATAGATGGC	641	Turton et al. [54]
WzxK5	F: TGGTAGTGATGCTCGCGA R: CCTGAACCCACCCCAATC	280	Turton et al. [54]
WzyK20	F: CGGTGCTACAGTGCATCATT R: GTTATACGATGCTCAGTCGC	741	Fang et al. [9]
WzxK54	F: CATTAGCTCAGTGGTTGGCT R: GCTTGACAAACACCATAGCAG	881	Fang et al. [9]
Wzy57	F: CTCAGGGCTAGAAGTGTCAT R: CACTAACCCAGAAAGTCGAG	1037	Pan et al. [41]
WzyK3	F: TAGGCAATTGACTTTAGGTG R: AGTGAATCAGCCTTCACCT	549	Fevre et al. [10]
*Bla*_KPC_	F-ATG TCA CTG TAT CGC CGT CT R-TTT TCA GAG CCT TAC TGC CC	538	Dallenne et al. [19]
*Bla*_IMP-1_	F-TGA GCA AGT TAT CTG TAT TC R-TTA GTT GCT TGG TTT TGA TG	139	Dallenne et al. [19]
*Bla*_IMP-2_	F-GGC AGT CGC CCT AAA ACA AA R-TAG TTA CTT GGC TGT GAT GG	139	Dallenne et al. [19]
*Bla*_VIM_	F-GAT GGT GTT TGG TCG CAT A R-CGA ATG CGC AGC ACC AG	390	Dallenne et al. [19]
*Bla*_NDM_	F-GGT TTG GCG ATC TGG TTT TC R-CGG AAT GGC TCA TCA CGA TC	521	Mushi et al. [24]
*Bla*_OXA-48 like_	F-TTG GTG GCA TCG ATT ATC GG R- GAG CAC TTC TTT TGT GAT GGC	281	Dallenne et al. [19]

### Recovery of isolates and carbapenem susceptibility

The isolates were transported to the microbiology laboratory, College of Veterinary Medicine Animal Resources and Biosecurity (COVAB) and instantly inoculated on blood agar for recovery. The identity of each isolate was reconfirmed by Microgen (Micro-biology International) kits for biochemical assays using procedures described by the manufacturer (www.microgenbioproducts.com). The isolates were subjected to antibiotic sensitivity assay on Muller-Hinton agar to ampicillin (AMP) 25 μg, amoxicillin/clavulanic acid (AMO) 20/10 μg, ciprofloxacin (CIP) 5 μg, cefuroxime (CXM) 30 μg, temocillin (TEM) 30 μg, piperacillin-tazobactum (TPZ) 110 μg, cefoxitin (FOX) 30 μg, cefipime (FEP) 30 μg, ceftriaxone (CRO) 30 μg, ceftazidime (CAZ) 30 μg, cefotaxime (CTX) 30 μg, ertapenem (ERT) 10 μg, imipenem (IMI) 10 μg and meropenem (MEM) 10 μg (Oxoid, UK). *E. coli* ATCC 25,922 was used as a susceptible strain and *Klebsiella pneumoniae* ATCC BAA-1705 as a positive control. Data generated by the susceptibility assay were interpreted based on the CLSI 2020 guidelines [16].

### Molecular characterization of *K. pneumoniae*

*Klebsiella pneumoniae* capsular molecular typing and characterization of carbapenem resistant genes was done by multiplex PCR employing adjusted methods used in the typing of *E. coli* by Toma et al*.* [17]. The first Multiplex PCR typing was based on primers targeting the K1, K2, K5, K20, K54, K57, and K3 capsular antigen genes to detect the major seven serovars [18], Table 1. The second multiplex PCR used primers targeting carbapenemase encoding genes namely; *bla*_VIM_, *bla*_IMP_, *bla*_KPC_, *bla*_OXA-48_, and *bla*_NDM_ [19], Table 1. Briefly, total genomic DNA was isolated using Qiagen DNA extraction kits according to the manufacturer’s instructions. For amplification, each multiplex PCR mixture contained a total of volume 50 μl composed of 23 μl 1X DreamTaqTM Green PCR Master Mix (Fermentas, Waltham, MA, USA), 0.8 μM of each primer pair (Eurofins Genomics AT GmbH, Austria) and 2.5 μl DNA template (100 ng/µl). Final PCR mixture volume was topped up to 50 μl and executed in a Bio-Rad PTC-200 Thermal Cycler (Bio-Rad, Hercules, CA, USA). For capsular typing, the PCR amplification conditions were; an initial denaturation at 95 °C for 5 min, then 35 amplification cycles at 95 °C for 30 s, 50 °C for 30 s, 72 °C for 1 min, and a final extension at 72 °C for 30 min. The reference strain *K. pneumoniae* GIM 46,117 (khe +) acted as a positive control while for carbapenemase genes molecular typing the annealing temperature was increased to 56 °C and the final elongation step performed for 10 min. PCR products were electrophoresed on a 1.5% agarose and stained with ethidium bromide to detect and assigning amplicons to their respective genes by comparing with 100–2000 base-pairs standard DNA ladder (Biomatik, USA). DSMZ 9377 *Klebsiella pneumoniae* was used as a negative control for all genes. *Klebsiella pneumonia* Nr.8 for NDM-1, *Klebsiella pneumoniae* 714 for OXA-48, *Klebsiella pneumoniae* 211 (T) for KPC, *P. aeruginosa* for IMP (Positive control strains from the Institute of Microbiology, Giessen, Germany) and *E. coli* for the VIM gene [20] were used as positive controls.

### Statistical analysis

Data analysis was performed using SPSS Version 25 statistical software. Chi square tests and Spearman’s correlation were used to compare the frequencies of carbapenem resistant isolates, carbapenem resistance genes, capsular serotypes and correlation of resistance genes to phenotypic resistance. A P-value of ≤ 0.05 signified substantial statistical variation.

## Results

*Klebsiella pneumoniae* isolates were obtained from different clinical samples of patients who were referred to the microbiology laboratories of the respective hospitals. A total of 284 isolates were obtained. However, 57 isolates were excluded because 24 isolates failed grow and 33 isolates were not *Klebsiella pneumoniae*. Of the 227 isolates used in this study, 128 were obtained from urine, 48 from pus swabs, 23 from blood, 16 rectal swabs, seven were from vaginal swabs, three from tracheal aspirate and two from sputum, Table 2.

**Table 2:** Distribution of *Klebsiella pneumoniae* isolates in different clinical samples

Hospital	Clinical sample							Total
	Urine	Blood	Pus swab	Rectal swab	sputum	Vaginal swab	Tracheal aspirate	
MNRH	44	6	19	5	2	5	1	82
MRRH	22	–	2	–	–	–	–	24
MBRRH	39	15	22	09	–	–	2	87
KIU-TH	23	2	5	2	–	2	–	34
Total	128	23	48	16	2	7	3	227

### Phenotypic carbapenem resistance profiles

Isolates used in this study were MDR as they exhibited resistance to different types of antibiotics, Table 3. Out of the 227 K*. pneumoniae* clinical isolates collected from different hospitals, 53 displayed phenotypic resistance to ertapenem; thus, this study established an overall phenotypic carbapenem resistance prevalence of 23.4%. Among the carbapenems, ertapenem registered the highest resistance (23.4%) while both imipenem and meropenem tied at 11.0%. Furthermore, MRRH scored the highest phenotypic carbapenem resistance prevalence (29.2%) followed by MBRRH (24.1%), MNRH (19.5%) and KIU-TH (11.8%), Table 3.

**Table 3:** Phenotypic antibiotics resistance profiles of *K. pneumoniae* clinical isolates obtained from different referral hospitals in Uganda

	Tertiary hospital				Total	Resistance prevalence (%)
	MNRH	MRRH	MBRRH	KIU-TH		
Number of isolates	82	24	87	34	227	–
AMP	82 (100%)	24 (100%)	87 (100%)	34 (100%)	227	100
AMO	82 (100%)	24 (100%)	85 (97.7%)	34 (100%)	225	99.1
SXT	82 (97.6%)	24 (100%)	85 (97.7%)	33 (97.1%)	222	97.8
CXM	81 (98.8%)	24 (100%)	86 (98.9%)	34 (100%)	225	99.1
TEM	80 (97.6%)	24 (100%)	80 (92.0%)	32 (94.1%)	216	95.2
TPZ	64 (78.1%)	19 (79.2%)	77 (88.5%)	25 (73.5%)	185	81.5
FOX	52 (63.4%)	14 (58.3%)	48 (55.2%)	15 (44.1%)	129	56.8
FEP	80 (97.6%)	24 (100%)	83 (95.4%)	33 (97.1%)	220	96.9
CRO	82 (100%)	23 (95.8%)	86 (98.9%)	34 (100%)	225	99.1
CAZ	82 (100%)	24 (100%)	86 (98.9%)	34 (100%)	226	99.6
CTX	82 (100%)	24 (100%)	87 (100%)	34 (100%)	227	100
CIP	30 (36.6%)	9 (37.5%)	30 (34.5%)	13 (38.2%)	82	36.1
ERT	17 (20.7%)	7 (29.2%)	25 (28.7%)	4 (11.8%)	53	23.4
IMI	11 (13.4%)	3 (12.5%)	7 (8.1%)	4 (11.8%)	25	11.0
MEM	11 (13.4%)	3 (12.5%)	7 (8.1%)	4 (11.8%)	25	11.0

### Distribution of carbapenemase encoding genes

**Fig. 1:**
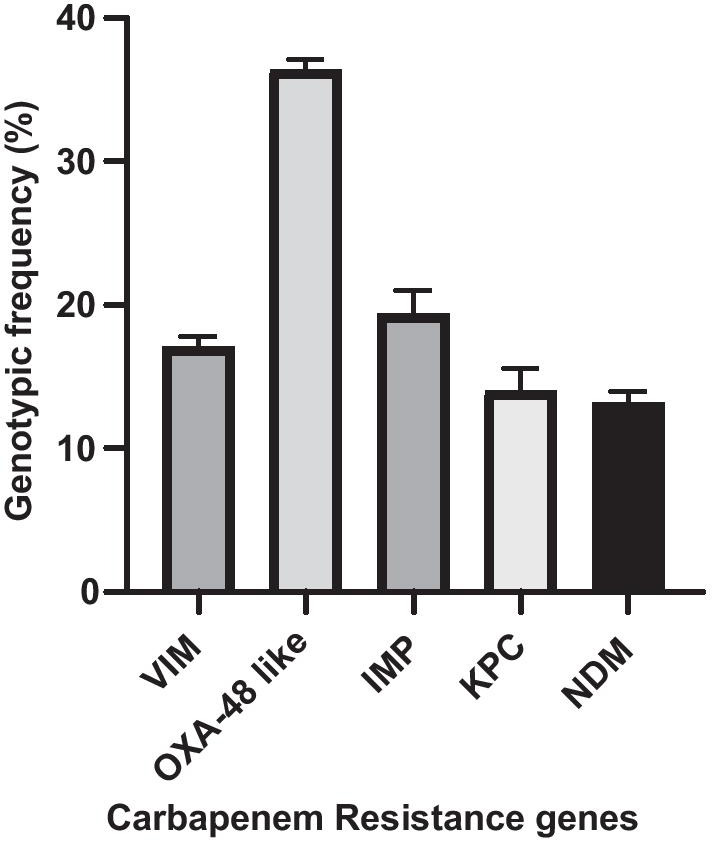
Genotypic frequency of carbapenemase encoding genes

From a total of 227 K*. pneumoniae* isolates, multiplex PCR amplification revealed that 43.1% (98/227) harbored one or more carbapenemases encoding gene combinations, Table 4. A total of 129 carbapenem resistance genes were scored. Of these, *bla*_OXA-48 like_ was the most predominant gene with a genotypic frequency of 36.4% (47/129) followed by *bla*_IMP-type_ (25/129 = 19.4%), *bla*_VIM-type_ (22/129 = 17.1%), *bla*_KPC-type_ (18/129 = 14.0%) and *bla*_NDM-like_ (17/122 = 13.2%). *K. pneumoniae* isolates obtained from MRRH scored the highest genotypic prevalence of carbapenem resistance (17/24 = 70.7%) followed by MNRH (35/82 = 42.7%), MBRRH (35/87 = 40.2%) and KIU-TH (11/34 = 32.4%), Tables 4 and 5, Fig. 1.

**Table 4:** Distribution of carbapenem resistant genes in *K. pneumoniae* isolates obtained from different referral hospitals in Uganda

Referral hospital	n	Carbapenemase encoding genes															
		NDM	KPC	IMP	OXA-48	VIM	NDM & OXA-48	KPC & IMP	KPC & OXA-48	IMP & OXA-48	VIM & OXA-48	VIM, NDM & OXA-48	NDM, KPC & OXA-48	IMP, NDM & OXA-48	NDM, KPC, VIM & OXA-48	Total CR isolates	Prevalence (%)
MNRH	82	3	3	8	8	2	2		2	–	–	2	2	2	1	35	42.7
MRRH	24	3	4	3	2		–	1	–	4		–	–			17	70.8
MBRRH	87	2	3	4	10	9	–	–	–		7	–	–	–	–	35	40.2
KIU-TH	34	–	2	3	5	1	–	–	–	–	–	–	–	–	–	11	32.4
Total	227															98	43.2

**Table 5:** Relationship between carbapenemase encoding gene with phenotypic resistance

Level of phenotypic and genotypic carbapenem resistance	Carbapenem resistance genes				
	VIM	OXA-48	IMP	KPC	NDM
Number of resistant isolates with the gene	22	18	15	13	7
Number of sensitive isolates with the gene	0	29	10	5	10
Percentage gene prevalence (%)	9.7	20.7	11.0	11	7.5
Percentage resistance conferred by gene presence (%)	100	38.3	60	72.2	41.2
Carbapenem resistance genotypic frequency (%)	17.1	36.4	19.4	14.0	13.2
Pearson Chi square P value	0.001	0.007	0.001	0.001	0.007

### Correlation of phenotypic resistance with genotypic resistance

Variation between phenotypic and genotypic was registered. The prevalence of VIM gene was 9.7% and conferred phenotypic resistance to 100% of the isolates that harbored it. This was followed by KPC-like which exhibited phenotypic resistance in 72.2% of the isolates, then IMP at 60%, NDM at 41.2% and then OXA-48 protected only 38.3% of isolates that housed it. All the carbapenemase encoding genes significantly correlated to phenotypic resistance with chi square P value < 0.05, Table 5

### Phenotypic carbapenem resistance profile among the *Klebsiella pneumoniae* pathotypes

The overall prevalence of pathogenic *Klebsiella pneumoniae* in Uganda was 46.3% (105/227) as revealed by multiplex PCR capsular typing. Of the 105 pathotypes, 37 exhibited phenotypic carbapenem resistance; thus, among the 53 phenotypic resistant *klebsiella pneumoniae* isolates, 37 (69.8%) were pathogenic. However, comparison of carbapenem resistance and susceptibility among the K pathotypes registered chi square P values > 0.05 indicating insignificant carbapenem resistance. PCR capsular typing targeted seven pathogenic genes where, WzyK5 scored the highest occurrence of 18.5% (42), followed by WzyK20 at 15.4% (35), WzyK3 at 7.1% (16), WzyK2 at 3.1% (07) and WzyK1 at 2.2% (05) Table 6. Capsular pathogenic genes WzyK54 and WzyK57 were not detected in any isolates. MRRH recorded the highest prevalence of *Klebsiella pneumoniae* pathotypes (15/24) trailed by MBRRH (46/87), KIU-TH (15/34) and MNRH (29/82). Pathogenic *Klebsiella pneumoniae* were isolated from urine (50), pus swabs (21), rectal swabs (19), blood (11), and other clinical specimens (04) Table 6.

**Table 6:** Phenotypic carbapenem resistance profiles of *Klebsiella pneumoniae* capsular pathotypes isolated from different clinical specimens

Pathotype	MNRH	MRRH	MBRRH	KIU-TH	Total capsular type/ Prevalence (%)	CR pathotypes	Chi square P value	Clinical specimen
WzyK1	2	1	2	0	5/2.2	2	0.12	Tracheal aspirate (2) and Rectal swab (3)
WzyK2	1	3	3	0	7/3.1	2	0.91	Blood (5) and Virginal swab (2)
WzyK3	5	4	7	0	16/7.1	6	0.31	Urine (13) and Rectal swab (3)
WzyK5	12	3	20	7	42/18.5	16	0.41	Urine (20), Pus swab (13), blood (3) and Rectal swab (6)
WzyK20	9	4	14	8	35/15.4	11	0.07	Urine (17), Pus swab (8), blood (3), rectal swab (7)
WzyK54	–	–	–	–	–	–	N/A	–
WzyK57	–	–	–	–	–	–	N/A	–
Total	29/82	15/24	46/87	15/34	105	37		
Prevalence %	35.4	62.5	52.9	44.1	46.7	16.3		

## Discussion

*Klebsiella pneumoniae* has been implicated as one of the main human pathogens causing nosocomial and community acquired infections over a long period of time. Due to antimicrobial resistance, treatment of *K. pneumoniae* infections has become exceedingly complicated (Moradigaravand et al. 2017). Furthermore, the situation is worsened when antimicrobial resistance is acquired by highly pathogenic strains. Most importantly, resistance to carbapenems in *Klebsiella pneumoniae* epitomizes a great threat to the delivery of health services worldwide. To decipher the state of affairs in Uganda, we investigated the prevalence of carbapenem resistant pathogenic *K. pneumoniae* in Uganda. Findings from this study exhibited that 56.4% of the MDR *K. pneumoniae* isolates were recovered from urine, 21.2% from pus swab and 10.1% from blood. Indeed, previous studies implicated *K. pneumoniae* as one of the predominant causes of urinary tract infections, surgical wound infections and bacteriemia [21, 22].

The study screened 227 MDR *K. pneumoniae* isolates obtained from four tertiary hospitals located in different regions for carbapenem resistance. High overall phenotypic carbapenem resistance prevalence of 23.3% was detected. This is in agreement with other studies in Uganda and Tanzania that reported phenotypic carbapenem resistance prevalence among Enterobacteriaceae of 22.4% [23] and 24% [24] respectively. However, a similar study at MBRRH detected lower phenotypic prevalence of 12.6% [25]. Contrary to this, studies in North Africa and West Africa reported remarkably higher phenotypic resistance of > 50% and *K. pneumoniae* were the most prevalent isolates [26–29]. Furthermore, a larger study which covered Gauteng, KwaZulu-Natal, Western Cape and Free State provinces in South Africa documented overwhelming phenotypic resistance of between 47 and 50% to imipenem, meropenem and doripenem, 84% and 89% to ertapenem [30, 31]. In comparison with previous studies at MNRH [23] and MBRRH [25] this study shows that the prevalence of carbapenem resistance in Uganda is on the rise and this is terrifying as recent meta-analyses revealed a substantial correlation between carbapenem resistant infections and increased risk of death [32].

Through molecular characterization, we detected carbapenem genotypic resistance frequency ranging from 32.4% at KIU-TH to 70.8% at MRRH and overall genotypic resistance prevalence of 43.2% in Uganda. In contrast, the overall genotypic prevalence was lower than that reported in Tunisia (86.3%) [33], Egypt (56%) [27], South Africa (86.0%), [31], India (76.3%) [34]. Among the five genes which were detected by multiplex PCR, the most encountered gene was OXA-48-like at a genotypic frequency of 36.4%. This corroborates well with recent studies which documented OXA-48-like gene and its variants as the most prevalent gene [27, 31, 33, 35]. OXA-48 was first detected in *K. pneumoniae* isolate in Turkey 2003. OXA-48 producers spread sporadically to the neighboring countries located in the southern and eastern part of the Mediterranean Sea, and north Africa [36]. This provides an insight why the occurrence of OXA-48 is predominantly high in Egypt and Tunisia [27, 33]. Previous studies reported NDM as the most dominant gene in South Africa [30, 37], VIM and IMP as the most prevalent genes in East Africa [23–25, 38] in contrast with the results of this study. This trend of events clearly shows that OXA-48 like producing *E. coli* and *K. pneumoniae* have invaded sub-Saharan Africa through immigration of individuals from the endemic region.

The overall phenotypic resistance registered by this study was lower than the genotypic resistance. For example, all isolates which harbored VIM expressed phenotypic resistance to ertapenem. Whereas OXA-48 like provided protection in only 38.3% of the isolates that sheltered it in disc diffusion assays. Oxacillinases encoded for by OXA-48 and its variant genes have been reported to possess low carbapenems hydrolyzing activity [36, 39, 40]. This enlighten why 61.7% of the isolates that housed OXA-48-like genes were sensitive to carbapenems. Furthermore, results of this study outlines that not all isolates that harbored carbapenemase genes were carbapenem insusceptible. This agrees with [40] findings who reported that modification and down regulation of outer membrane proteins through which drugs diffuse to reach their targets complements gene products and among the carbapenems, ertapenem is affected most by this scenario. This elucidates why resistance to ertapenem was significantly high. Thus, presence of a carbapenemase encoding genes alone does not guarantee resistance.

The capsule is one of the major factors that influence virulence in *K. pneumoniae*. Several studies have documented how capsular types influence pathogenicity of *K. pneumoniae* associated infections [9, 41]. Previous studies unraveled the structures of the gene cluster in *Klebsiella spp* responsible for capsular polysaccharide synthesis (CPS) in some types [42, 43]. The genetic structure is composed of a cluster of six highly conserved genes among different capsule types namely galF, cpsACP, wzi, wza, wzb and wzc that encodes for proteins that play a role in CPS translocation and processing at the bacterial surface and are located at the 5′ end of the cps regions and genes encoding glucose-6-phosphate dehydrogenase (gnd) and UDP-glucose dehydrogenase (ugd) found at the 3′ end. In the middle of the CPS loci lies a variable region that contains certain genes (Wzy and Wzx) that transcribe proteins accountable for polymerization and putting together of the specific CPS subunits. Thus, the great sequence variation of the wzy gene among the different capsular types is the basis of PCR capsular typing assays [43–45]. In light of this, we exploited the Wzy gene to characterize the most clinically important *K. pneumoniae* capsular serotypes isolated from different tertiary hospitals in Uganda.

Capsular typing by heptaplex PCR revealed that K1, K2, K3, K5 and K20 accounted 46.7% (106/227) of the *K. pneumoniae* clinical isolates. K54 and K57 were not detected in any of the isolates. *Klebsiella pneumoniae* K1 and K2 have been reported as the most virulent capsular types causing septicemia and liver abscess [41, 43]. However, other capsular serotypes are equally important as K5 and K20 are also associated with community acquired ailments whereas K3 causes chronic granulomatous infection of the nasal cavity and in some patients, the infection advance and lead to severe respiratory impairment [10, 13]. Thus, the high prevalence of pathogenic capsular serotypes isolated from clinical specimens is a great threat to the healthcare system. There is no data about incidence of *K. pneumoniae* K types within the sub-Saharan region for comparison. However, results of this study are in line with Lin et al. [46], who reported K1, K2, K3, K5 and K20 as the most prevalent capsular types in Taiwan. Furthermore, out of the 106 *klebsiella pneumoniae* capsular types, 37 exhibited resistance to carbapenems yet carbapenems are regarded as the drugs of choice for treatment of MDR Gram-Negative HAI when the first line drugs have failed [47]. Acquisition of carbapenem resistance in pathogenic bacteria correlates with treatment failure in addition to increased morbidity and mortality [21]. Investigations elsewhere which looked clinical samples, associated coexistence of capsular and other virulent factors such as rmpA and aerobactin genes with hypervirulent or hypermucoviscous *K. pneumoniae* variant (hvKP) [48, 49]. Despite the fact that this study did not attempt to detect other virulence factors, high occurrence of carbapenem resistance in capsular serotypes detected in study suggests possible existence carbapenem resistant hypervirulent *K. pneumoniae* (CR-HvKP) in Uganda clinical settings. Indeed, this has been case in clinical settings with substantial carbapenem resistance [50–52].

## Conclusion

Findings of this study show that clinical *K. pneumoniae* isolates obtained from representative tertiary hospitals in Uganda exhibit high diversity of the main virulent capsular types and antibiotic resistance profiles to the frontline and last resort antibiotics. Based antimicrobial susceptibility assay, PCR capsular and carbapenemase gene typing, substantial prevalence of highly virulent MDR *K. pneumoniae* isolates were present in clinical specimens. High incidence of such isolates poses great health risks within healthcare and community settings; thus, should be treated with urgent attention. To the best of our knowledge, this is the first study to unravel the carriage of carbapenem resistance in pathogenic *K. pneumoniae* clinical isolates in Uganda. Thus, our data informs the need for regular surveillance of antibiotic resistance in pathogenic bacteria in clinical settings for meaningful control of emergence and spreading of AMR pathogens. Furthermore, this study only investigated carbapenem resistance carriage in capsular serotypes. However, to provide an insight into carbapenem resistance carriage in all *K. pneumoniae* pathotypes, additional virulence genes such as the rmpA gene expressing regulator of mucoid phenotype A; allS gene which is translated into the activator of the allantoin regulon, connected to allantoin metabolism; endotoxin encoding genes wabG, uge, and wcaG; iron acquisition system codifying genes iucB, iroNB, ybtA, and kfuBC; adhesin gene fimH (type I fimbriae); and ureA gene coding for a-subunit of the urease should be characterized.

## Data Availability

All data generated by this study have been submitted with this manuscript. Raw data and any other forms data generated by this project can be obtained from the authors on request by e-mail.

## Abbreviations

MDR: Multidrug-resistant
HAI: Healthcare associated infections
UTI: Urinary tract infection
K: Capsular
MNRH: Mulago National Referral Hospital
MRRH: Mbale Regional Referral Hospital
MBRRH: Mbarara Regional Referral Hospital
KIU-TH: Kampala International University-Teaching Hospital
COVAB: College of Veterinary Medicine Animal Resources and Biosecurity
AMP: Ampicillin
AMO: Amoxicillin/clavulanic acid
CIP: Ciprofloxacin
CXM: Cefuroxime
TEM: Temocillin
TPZ: Piperacillin-tazobactum
FOX: Cefoxitin
FEP: Cefipime
CRO: Ceftriaxone
CAZ: Ceftazidime
CTX: Cefotaxime
ERT: Ertapenem
IMI: Imipenem
MEM: Meropenem
ATCC: American Type Cell Cultures
*bla*: Beta lactamase
KPC: *Klebsiella pneumoniae* Carbapenemase
NDM: New Delhi Metallo-β-lactamase
VIM: Verona Integron-encoded Metallo-βlactamase
IMP: Imipenemase Metallo-β-lactamase
OXA: Oxacillinase
CPS: Capsular polysaccharide synthesis
